# Draft Genome Sequence of Zygosaccharomyces mellis CA-7, Isolated from Honey

**DOI:** 10.1128/MRA.00449-19

**Published:** 2019-06-27

**Authors:** Yuh Shiwa, Yu Kanesaki, Taichiro Ishige, Kiyoshi Mura, Taichi Hori, Tomoko Tamura

**Affiliations:** a Department of Molecular Microbiology, Tokyo University of Agriculture, Setagaya, Tokyo, Japan; b NODAI Genome Research Center, Tokyo University of Agriculture, Setagaya, Tokyo, Japan; c Department of Nutritional Science and Food Safety, Tokyo University of Agriculture, Setagaya, Tokyo, Japan; University of California, Riverside

## Abstract

In this study, we report the draft genome sequence of Zygosaccharomyces mellis CA-7, isolated from purchased honey imported from Canada. The 10.19-Mb genome contains 4,963 gene models. To our knowledge, this annotated genome sequence is the first from the species *Z. mellis* and will contribute to a better understanding of the osmotolerance of microorganisms in high-sugar products.

## ANNOUNCEMENT

Osmotolerant microorganisms can survive in high-salt/sugar environments. Zygosaccharomyces mellis and Zygosaccharomyces bailii can grow in preserved food such as honey and maple syrup (1). Zygosaccharomyces rouxii can grow in soy sauce and miso, which are traditional fermented foods in Japan that contain large amounts of salt (2). *Z. mellis* is known to deteriorate the quality of honey (3).

We isolated *Z. mellis* from purchased honey imported from Canada using M40Y medium (2% malt extract, 0.5% yeast extract, and 40% glucose) and named it CA-7. The strain was able to grow in yeast malt (YM) medium (0.3% yeast extract, 0.3% malt extract, and 0.5% polypeptone) with 60% glucose, in which Saccharomyces cerevisiae cannot survive.

To obtain the draft genome sequence of *Z. mellis* CA-7, the DNA was extracted using the benzyl chloride method (4). In addition, we performed transcriptome sequencing (RNA-seq) analysis to obtain information on gene expression in hyperosmotic medium. *Z. mellis* was cultured in YM medium containing 50% glucose (pH 5.0) or 1% glucose (pH 5.0) at 28°C for 12 hours on a shaker at 160 rpm. Cells were then spheroplasted with a buffer containing zymolyase at a final concentration of 25 U/1 × 10^7^ cells. RNA was extracted from the spheroplasts using the RNeasy minikit (Qiagen, Redwood City, CA) following the manufacturer’s instructions.

Genomic DNA was sequenced using the Illumina Genome Analyzer IIx platform and a paired-end read library with an insert size of 500 bp, producing 43,801,006 104-bp paired-end reads. The paired-end reads were subjected to quality trimming (removal of adapter sequences and low-quality sequences with a quality limit of 0.05, two ambiguous nucleotides allowed per read, and a minimum read length of 50 bp) and assembled using CLC Genomics Workbench (Qiagen) version 11.0.1 with default parameter settings. The assembly consists of 1,643 scaffolds, 85 of which are larger than 1 kb, with an *N*_50_ value of 271,063 bp, comprising 10.19 Mbp in total, and a coverage of 447×. The average G+C content is 38.3%. Only scaffolds larger than 1 kb were retained for further annotation and deposited.

In addition, RNA-seq was carried out in the above-mentioned medium. cDNA sequencing libraries were prepared from 100 ng of total RNA using a TruSeq RNA sample prep kit (Illumina) according to the manufacturer’s instructions. A total of 280.9 million paired-end sequence reads of 100 bp (total, 27.9 Gbp) were generated using an Illumina HiSeq 2500 instrument.

Genome annotation was performed with the Funannotate pipeline version 1.4.2 (http://www.github.com/nextgenusfs/funannotate), which includes repeat masking, training of *ab initio* gene predictors with RNA-seq data, and annotation steps. In total, 4,963 protein-coding gene models were annotated, which is consistent with the predictions for Z. rouxii CBS 732, belonging to the same genus (5). Completeness of the predicted proteins was assessed using BUSCO version 3.0 (6), which showed the presence of 96.0% of *Saccharomycetales* sp. single orthologous genes (saccharomycetales_odb9). To the best of our knowledge, this annotated genome sequence is the first from *Z. mellis* and will contribute to a better understanding of the osmotolerance of microorganisms in high-sugar products.

### Data availability.

This whole-genome shotgun project has been deposited in DDBJ/EMBL/GenBank under the accession no. BIMX01000001 to BIMX01000085. The raw Illumina data have been deposited under accession no. DRA008319. The version described in this paper is the first version.
